# Development and testing of material extrusion additive manufactured polymer–textile composites

**DOI:** 10.1186/s40691-020-00232-7

**Published:** 2021-01-05

**Authors:** Giselle Hsiang Loh, Adeayo Sotayo, Eujin Pei

**Affiliations:** 1 PhD Candidate, Department of Design, Brunel University London, Kingston Lane, Uxbridge, UB8 3PH UK; 2 Research Fellow, Department of Design, Brunel University London, Kingston Lane, Uxbridge, UB8 3PH UK; 3 Senior Lecturer, Department of Design, Brunel University London, Kingston Lane, Uxbridge, UB8 3PH UK

**Keywords:** 3D printed textiles, Material extrusion, Polymer–textile composite, Polymer–textile adhesion, Mechanical testing, Adhesion testing, T-peel test

## Abstract

The adoption of Additive Manufacturing (AM) has gradually transformed the fashion industry through innovation and technology over the last decade. Novel AM systems and techniques are continuously being developed, leading to the application of AM polymers with textiles and fabrics in the fashion industry. This work investigates the development and testing of polymer–textile composites using polylactic acid (PLA) filaments on synthetic mesh fabrics using direct material extrusion (ME). An aspect of this paper highlights the appropriate combination of printing material, textile substrate, and printer settings to achieve excellent polymer–textile adhesion. Details of the printing process to create polymer–textile composites are described, as are the interfacial strength results of the T-peel tests, and the observed failure modes post-testing. The peel strengths for different ME bonded polymer–textile composites are examined and used to identify the compatibility of materials. This work visualised the potential of direct ME of polymers onto textile fabrics as a material-joining approach for new textile functionalisation, multi-material composite explorations and innovative aesthetic print techniques. This work also adds to the limited knowledge of AM polymer–textile composites, which can provide helpful information for designers and researchers to develop new applications and facilitate future research development in smart embedded and programmable textiles.

## Introduction

Additive Manufacturing (AM) commonly known as 3D Printing or Rapid Prototyping enable the fabrication of geometrically complex components by precisely placing material(s) one layer at a time in position within a design domain. In general, the benefits of AM include design freedom, low tooling start-up cost, rapid verification with reduced time to market in product development, service and increased R&D efficiency (AM Platform 2014; Redwood et al. 2017). AM is also constantly progressing with future perspectives in hardware, software, and materials to expand the potential of prototyping and applications across different industries including the textile, aerospace, construction, pharmaceutical, and biomedical sectors. Recently, AM was instrumental in the fight against COVID-19, through the development of personal protective equipment (PPE) and other medical equipment (e.g. test swabs, ventilators) (Singh et al. 2020).

More precisely, the rise in the adoption of AM has led to a significant transformation of the fashion and textile industry through innovation and technology. One of the pioneers of this adoption is the influential designer, Iris Van Herpen. In 2010, she showcased her first 3D printed dress, which led to greater awareness and exploitation of the technology being employed in the fashion industry (Van Herpen 2010). The role of AM has continually evolved with increasing awareness and interest in the technology from researchers and designers. The number of research publications on “3D Printing Textiles” has continuously increased over the past few years with over 4000 publications on Google Scholar in 2019. This figure shows that AM will potentially open up new opportunities in fashion and textile innovation, promoting localised production of on-demand and personalised garments, allowing smaller batches or home production to compete in the market (Table 1).

**Table 1:** Examples of applications and future research direction for ME polymer–textile composite

Development	Application	Brand/subject	Description	Refs.
Product	Wearables	LabeledBy; Tamicare	Personalised, localised, and sustainable garments and fabrics	LabeledBy (2020), Lopez (2020), Tamicare (2020)
	Mounting or embossing elements	Braille on textiles	Modifications of textile surface properties to support blind people	Kreikebaum et al. (2017)
	Orthopaedic devices	Glove; knee brace	Customised orthopaedic devices	Ahrendt and Karam (2020), Uysal and Stubbs (2019)
Research	Programmable or stimulus-responsive textiles (4D printing)	Hybrid textiles	Polymer–elastic textiles composite: the elastic textile is pre-stretched prior to printing, the stored energy in the textile material prior to printing causes a change in form when the energy is released	Narula et al. (2018), Papakonstantinou (2015)
		Shape change and self-assembly	Stimulus-responsive polymer–textile composite: stimulus-responsive textiles that that can self-transform or morph from one form to another when subject to an external stimulus	Leist et al. (2017), Momeni et al. (2017), Zapfl (2019)
	Textile-based sensors or electronics	Self-sensing or actuator	Conductive materials or biohybrid materials—textile composite: sensing body and sensing element	BioLogic (2015), Gehrke et al. (2019), Kumar et al. (2019)

The combination of digital manufacturing techniques gives the possibility for a textile to be three-dimensionally manufactured without tedious labour work, complex pattern-cutting, stitching, or the use of a specific mould. This approach also promotes a more environmental conscious and sustainable future for materials used in the fashion industry (Flynt 2019; Kim et al. 2019; Mageean 2018; Van der Velden et al. 2015; Zapfl 2019). However, the production of AM textiles is machine-intensive which require extensive understanding of the materials, the design and modelling programs, and the printing production process.

This paper focuses on the development and testing of material extrusion (ME) AM polymer–textile composites, which involves direct printing of thermoplastics onto conventionally manufactured textile fabric substrates. This novel material-joining technique highlights the synergy between conventional manufacturing processes and AM process to encourage a new vision of polymer–textile functionalisation and multi-material exploration in the textile industry. This study contributes to new knowledge and understanding of ME polymer–textile composites to facilitate future research development and integration of novel AM technology in textile design. For instance, Functionally Graded Additive Manufacturing (FGAM) with the integration of digital materials using PolyJet technology can offer sophisticated localised graded colours and different properties on a single piece of textile by varying the material organisation at a precisely defined area (Bader et al. 2016; Loh et al. 2018; Oxman 2011). New materials such as shape-memory materials can be used to create programmable or stimulus-responsive textiles that can transform or morph from one form to another when subjected to an external stimulus, known as 4D Printing (Leist et al. 2017; Pei and Loh 2018).

This paper discusses three key interconnected factors (i.e. printing material, textile substrate and printer settings) affecting the production and overall quality of the polymer–textile composites. This paper also gives details of the manufacturing process, as well as the experimental setup, procedures, and analysis techniques used to quantify the adhesion properties for different orientations of bonded ME printed polymer–textile composites. This study investigates the effect of varying textile substrate parameters (i.e. different fibre types, structure, and weights) on the polymer–textile adhesion force. The printing material used, and ME printing parameters were kept constant. Different combination of ME printed polymer–textile composites using PLA (printing material) and Nylon and Polyester (textile substrates) were manufactured to evaluate their manufacturing feasibility and assess their mechanical properties. The manufacturing demonstration and experimental results adds to the current limited knowledge of developing and testing of ME printed polymer–textile, which provide useful information for designers and researchers to facilitate further research and increased uptake towards industry-wide applications.

## Factors affecting ME printed polymer–textile composites

**Fig. 1:**
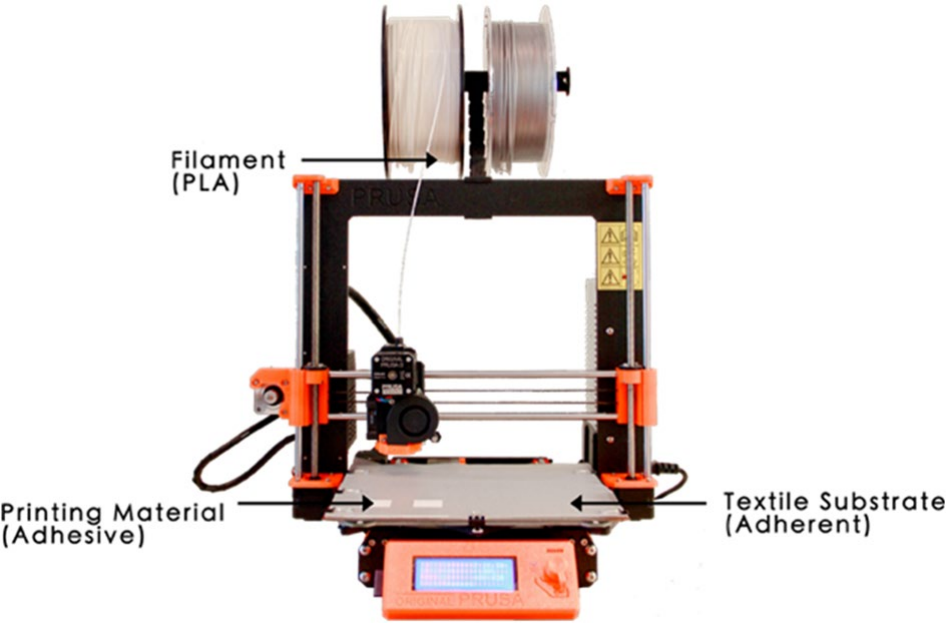
Desktop ME printer (Original Prusa i3 MK3S 3D Printer) setup for ME onto the textile substrate

ME as a category of AM process described in ISO-ASTM 52900 (ISO/ASTM 2017), often known as “Fused Deposition Modelling (FDM)” and “Fused Filament Fabrication (FFF)” is the predominant method of manufacturing polymer–textile composites (Chatterjee and Ghosh 2020). The AM process involves the material from a spool of filament loaded into the printer, melted above its glass transition temperature (T_g_) for amorphous polymers and above its melt temperature (T_m_) for semi-crystalline polymers. The polymer is then selectively dispensed through the heated extrusion nozzle and deposited onto the build platform at a predetermined location (Loh et al. 2020; Redwood et al. 2017). This technology of additively building up material by selectively dispensing through a nozzle or orifice allows AM parts to be built directly on the surface of the textile substrate. Sanatgar et al. (2017) described it as a thermal welding method for joining of the printing material (adhesive) and the textile substrate (adherent) during the ME process (see Fig. 1).

There are three major interconnected factors that affect the fabrication, polymer–textile adhesion and the overall quality of ME polymer–textile composites (Loh and Pei 2019; Melnikova et al. 2014; Pei et al. 2015).

- Printing material,
- Textile substrate, and
- Printer settings.

### Printing material

ME makes use of thermoplastics in the form of filaments, which are typically 1.75 mm or 3 mm in diameter (Redwood et al. 2017). ME processes allow a wide variety of materials with diverse characteristics and properties to be used, ranging from commodities, engineering, to high-performance thermoplastics, composites, and functional materials (Loh et al. 2020). Table 2 gives details of some of the common thermoplastics used in ME, their key material characteristics, cost as well as their printing parameters including the nozzle and build platform temperature. The printing temperature, performance and cost of the materials increase through each material category from PLA to Polyetherimide (PEI) (Redwood et al. 2017; Rigid.Ink 2019). In general, the better the engineering properties of thermoplastics, the higher the temperature required to heat the material to a deformable state, and therefore, the more difficult the material is to print (Redwood et al. 2017). The use of materials with lower printing temperature is recommended to avoid damaging or burning the textile substrate during direct deposition of the polymer.

**Table 2:** Common thermoplastics used in ME and their properties

Material type	Filament material	Material characteristics	Cost (£/kg)	Nozzle temperature (°C)	Build platform temperature (°C)	Refs.
Commodity	PLA	Biopolymer, lower impact strength and temperature resistance	32	180–210	20–45	Rigid.Ink (2019), Tyson (2020c)
	PLA Plus+	Very durable biopolymer, vibration absorbing and less brittle version of PLA	37	220–230	50–60	Gregurić (2020), Rigid.Ink (2019)
	Flexible PLA	Flexible and durable biopolymer, good vibration dampening	37	240–250	30–60	Griffin (2019), Rigid.Ink (2019)
	ABS	Strong and durable, good temperature resistance but susceptible to warping	32	230–250	90–95	Rigid.Ink (2019), Tyson (2020e)
Engineering	PETG	Extremely durable, high impact and chemical resistance, low shrinkage	40	220–245	70–80	Rigid.Ink (2019), Tyson (2020b)
	TPU	Flexible and rubber-like, stretchy properties with good elongation but difficult to print accurately	49	210–240	20–70	Rigid.Ink (2019), Tyson (2020d)
	Nylon (PA 12)	Extremely durable, flexible, low friction for high impact and high stress prints	38	255–275	100–110	Rigid.Ink (2019), Tyson (2020a)
High performance	PEI	Excellent strength to weight, fire, and chemical resistance	250	355–390	120–160	3D^4^Makers (2020)

### Textile substrate

Table 3 describes the variables of the textile substrates that affect the polymer–textile adhesion of ME printed polymer–textile composites. These properties include, but not limited to, the types of fibres, fabric weight, weave pattern, weft density and surface properties. These variables determine the type of print structure layout appropriate for the chosen textile substrate proposed in Table 4. Print layout A involves embedding the textile substrate between two print layers to form a laminated composite, while print layout B involves a one-sided print, deposited directly on the textile substrate. Print layout A is suitable for printing on “open” mesh or perforated textile substrates, whereas print layout B is suitable for “closed” tightly woven textile substrates (Meyer et al. 2019).

**Table 3:** Different variables of textile substrates that can affect the polymer–textile adhesion

Textile substrate properties and structure	List of variables	Options	Characteristics or descriptions	Refs.
Fibre types	Plant	Cotton	Cool, soft, and comfortable; absorbs and releases respiration quickly; durable but wrinkles easily	Elliot (2015), Korger et al. (2016), Mpofu et al. (2019), Pei et al. (2015)
		Linen	Woven from the stems of flax; two-times stronger than cotton; absorbs and releases perspiration quickly; lightweight; non-stretchable and wrinkles easily	
	Animal	Wool	Ranges from scratchy to very soft; absorbs 30% of its weight in moisture; absorbs and releases moisture quickly; dirt and flame resistant; stronger when dry; performs as an insulator	
		Silk	Versatile, soft, and comfortable; strongest natural fibre; absorbs and releases perspiration quickly; easily dyed; retains shape and drapes well but weakened by sunlight and perspiration	
	Synthetic	Rayon	Strong; extremely absorbent; soft and comfortable; made in a variety of qualities and weights but wrinkles easily	
		Acetate	Crisp and soft; suitable for dyes and prints; shrink, moth and mildew resistant; low moisture absorbency and fast drying	
		Nylon	Strong, lightweight, stretchable, and durable; dries quickly; easy to clean; resistant to abrasion and chemicals; does not absorb moisture well	
		Acrylic	Lightweight, soft, and warm; dyes to bright colours; absorbs and releases moisture quickly; retain shape and resists shrinkage and wrinkles; hold pleats; resistant to moths, oils and chemical, and sunlight degradation	
		Polyester	Strong, stretchable, and durable; does not wrinkle; dries quickly; does not absorb moisture	
Weight	Denier	Low denier count	Denier is a method for measuring the fineness of fibres, defined by the mass in grams per one strand of 9000 m fibre. High denier count fabrics tend to be thick, sturdy, and durable while low denier count fabrics tend to be sheer, soft, and silky	Hindman (2013a), Standard Fiber (2020)
		High denier count		
	Stitch density	Low stitch density	Stitch density is a measurement of the number of stitches per inch (SPI) of fabric as it passes from the entrance of a needle loom to the exit	Hindman (2013b)
		High stitch density		
	Weft density	Low weft density	Warp and weft are the two basic components used in weaving to turn thread or yarn into fabric. The adhesion force decreases when weft density increase	Malengier et al. (2017), Mpofu et al. (2019, 2020), Narula et al. (2018)
		High weft density		
	Warp linear density	Low warp linear density	The adhesion force increases when the linear density increase	
		High warp linear density		
	Pore properties	Fine	The pore properties include the pore size, pore size distribution, pore shape, and porosity determined by the fibre properties and structural properties, such as setting and weave type	Eutionnat-Diffo et al. (2019), Ragab et al. (2017)
		Large		
Surface	Finish	Mechanical	Squished, Circe’ finish, brushed or knapped	Korger et al. (2016), Meyer et al. (2019), Unger et al. (2018)
		Chemical	Polymer coating (i.e. PMMA coating), plasma treatment	
		Washing	Washing agent, enzyme amylase	
	Texture	Surface appearance	Texture is defined by the surface appearance, structure, and thickness of the fabric. Texture is created by the fibre type, by weaving or knitting process, or by fabric finishes. Examples of textures include fuzzy, furry, soft, shiny, dull, bulky, rough, crisp, smooth, and sheer	Sew Guide (2020)
		Structure		
		Thickness

**Table 4:** Types of print layout

Print layout A	
Print layout B	

### Printer settings

Desktop Cartesian ME printer uses a system of X–Y–Z coordinates to determine the location of the extrusion nozzle, which allows direct ME onto the textile substrate. The setup is shown in Fig. 1 using an Original Prusa i3 MK3S ME machine with a single extruder. ME machines with multiple extruders can be used to create multi-material AM components, achieved by swapping the filament materials at a predetermined location or between layer changes.

Table 5 identifies some of the ME processing parameters taken into consideration and the settings used during the fabrication of polymer–textile composites. These results are based on preliminary tests and literature review. The processing parameters include the Z-distances, printing temperature (Table 2), layer height, printing speed, fill settings, extrusion width, flow rate as well as build platform surface. The printer settings have a great impact on the visual and haptic finishing of the printed structure (Pei et al. 2015). The Z-distance has a significant effect on the adhesion of polymers to the textile substrate and quality of the print. An increment in Z-distance (build platform to extrusion nozzle adding fabric thickness) must be applied while printing on mid-weight to heavy-weight or textured textile substrate to compensate the fabric thickness. An optimum Z-distance adjustment should prevent the extruder nozzle from getting caught on the fabric but close enough to press the extruded polymer into the textile substrate with no gaps between deposited parameters. In line with an optimum Z-distance between the nozzle and the build platform used, 0.1 mm and 0.2 mm layer height can usually provide good linear surface finishing with no scars on the top surface, messy first layer or gaps between infill and outline (Loh et al. 2020). A layer height greater than 0.2 mm exhibited a negative effect on dimensional accuracy and adhesion force (Spahiu et al. 2017). The printing temperature and printing speed have the largest effect on the adhesion force (Sanatgar et al. 2017). High nozzle temperature can reduce the material viscosity, allowing deeper and stronger material penetration into the textile substrate (Spahiu et al. 2017). For printing taller or larger components, the nozzle temperature can be adjusted back to the suggested temperature after five print layers on top of embedded textile to prevent overheating. Although Sanatgar et al. (2017) claimed that the build platform temperature does not affect the adhesion force, an optimum build platform temperature can provide better first layer adhesion to build platform and prevent warping. The extrusion width should be set at 100% or 150% greater than the default nozzle diameter (> 0.4 mm) in order to generate enough material to penetrate into the textile fabric (Spahiu et al. 2017). The study by Spahiu et al. (2017) also revealed that increasing the printing speed and polymer flow rate showed no substantial effect on the polymer–textile adhesion.

**Table 5:** The list of ME printer settings taken into consideration during the fabrication of the polymer–textile composites

Printer parameters	List of variables	Settings or suggestions	Refs.
Z-distance	Build platform to extrusion nozzle	Calibrate the optimum Z-height through first layer calibration	Grimmelsmann et al. (2018), Prusa^3^D (2018)
	Build platform to extrusion nozzle adding fabric thickness	Multiple first layer calibration and preliminary printing tests results given that the optimum Z-height increment is by adding halved of the fabric thickness. (i.e. increment between + 0.05 mm and + 0.07 mm for fabric thickness of 0.15 mm). This is applicable when using mid-weight to heavy-weight and textured textile substrates	Sanatgar et al. (2017), Spahiu et al. (2017)
Printing temperature	Nozzle temperature	Increase 5 °C to 10 °C on top of suggested temperature by manufacturer	
	Build platform temperature	As suggested by manufacturer	
Layer height	First layer	0.2 mm	
	Subsequent layer	0.1 mm	
Printing speed	First layer	20 mm/s	
	Perimeters	45 mm/s	
Fill	Pattern	Rectilinear	
	Angle	0°; solid infill threshold area 90°	
	Density	100%	
Extrusion width (nozzle diameter: 0.4 mm)	First layer	0.42 mm	
	Subsequent layer	0.45 mm	
Flow rate	N/A	100%	
Surface	Build platform	PEI sheet, blue painter’s tape, Build Tak, flex plate, Magigoo or heated glass	Loh et al. (2020)

## Methods

### Materials and AM process

This work explains the procedure of direct ME off-the-shelf PLA on selected mesh fabrics using print layout A. Three different combinations of polymer–textile–polymer composites were produced as shown below:

- PLA—Nylon (net structure)—PLA,
- PLA—Polyester (voile structure)—PLA, and
- PLA—Nylon (voile structure)—PLA.

The printing material was Prusa PLA filament with a diameter of 1.75 mm. PLA is cost-effective and relatively easier to print at a lower nozzle and build platform temperature, without burning the textile substrates. This is because PLA has a melting point of 260 to 270 °C (Callister and Rethwisch 2018). It has relatively low warping and stringing properties, leading to high detail finishing and better overall aesthetical quality (Tyson 2020c). The three different types of textiles substrates used were namely Nylon (net structure), Polyester (voile structure) and Nylon (voile structure). Table 6 gives the properties of the three lightweight mesh fabrics. The woven Polyester and Nylon voile shared relatively similar properties, comprising fabric thicknesses, non-stretch properties, fine pore sizes with smooth and sheer surface texture. On the other hand, the knitted Nylon net fabric had larger thicknesses (almost double), stretchable horizontally, relatively larger pore sizes of approximately 2 × 1.5 mm with rough surface texture.

**Table 6:** Properties of the three textile substrates (mesh fabrics) used for the ME polymer–textile composites

Name	Structure	Process	Thickness (mm)	Pore size	Stretch	Melting point (°C)	Ref.
Nylon	Net	Knitted	0.25	Large	One-directional (horizontally)	260–270	Callister and Rethwisch (2018)
Polyester	Voile	Woven	0.13	Fine	Non-stretch	260–270	
Nylon	Voile	Woven	0.14	Fine	Non-stretch	260–270	

**Fig. 2:**
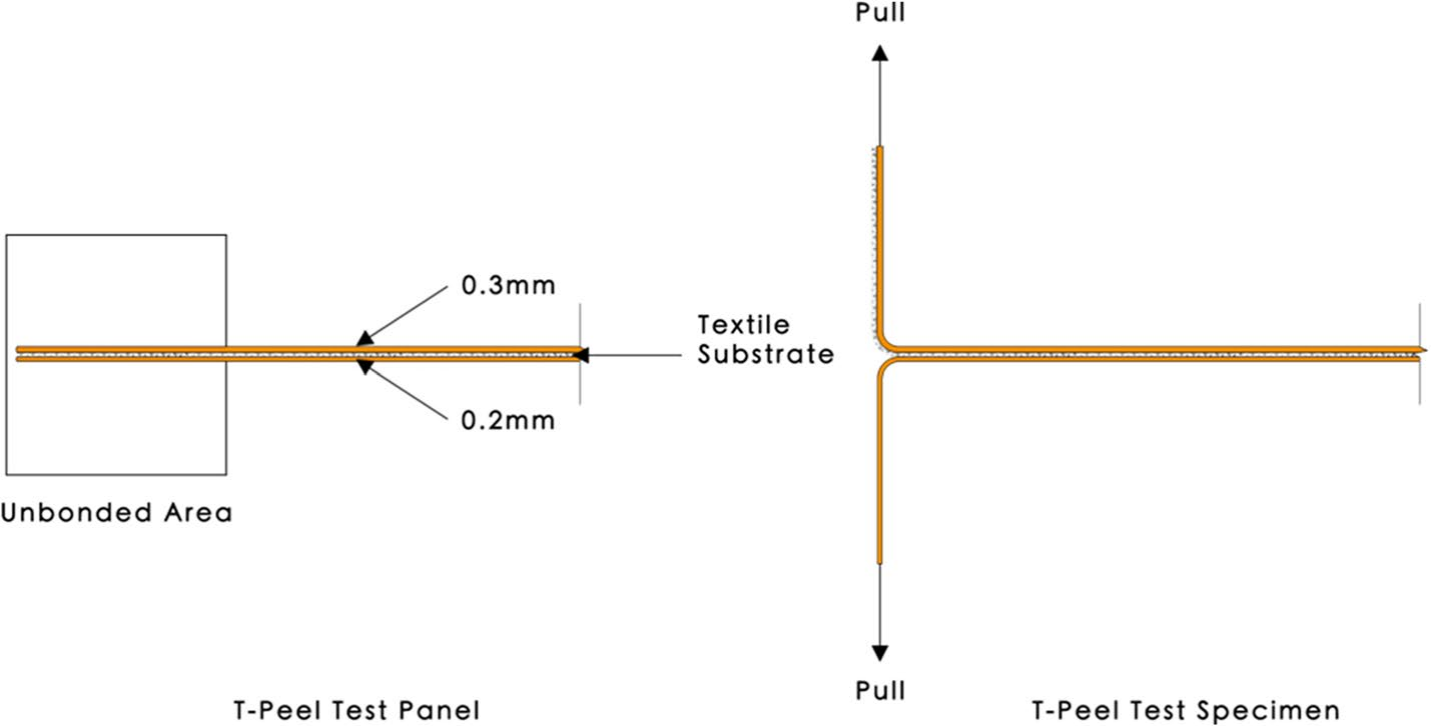
Illustration of the ME polymer–textile composite for the T-Peel test (not drawn to scale)

Adhesion affects the durability and quality of the final product (i.e. polymer–textile composite). Therefore, it was deemed appropriate to investigate the mechanical properties (via T-peel tests) of the bonded ME polymer–textile composites, to determine the optimum printing material and textile substrate combination and orientation. The ME polymer–textile composites designed for the T-Peel test were in line with British Standards (BS) EN ISO 11339 (2010) (Fig. 2). To manufacture the polymer–textile composites using ME, the CAD design of the printed structure (L × W × H of 200 mm × 150 mm × 0.5 mm) was created using SolidWorks, exported as an STL.file, imported into Slic3r for slicing and exported as a G-code for printing. The polymer–textile composites were manufactured using an Original Prusa i3 MK3S 3D Printer with a 250 mm by 230 mm build platform and a nozzle diameter of 0.4 mm, using the printer settings specified in Table 5. The nozzle temperature to print Prusa PLA was 220 °C, while the build platform temperature was set at 60 °C.

As far as the printing procedure for ME polymer–textile composites (print layout A) was concerned, the first or base PLA layer (0.2 mm layer height) was printed on the build platform using the calibrated Z-distance between build platform to extrusion nozzle. While the first layer print was almost complete, the nozzle height was increased to a new Z-distance to offset the fabric thickness. The printer was paused immediately once the first layer was completed. The Z-axis will automatically be lifted from the build platform by the system (Table 7A). To create the T-Peel test panel, a section of blue tape was applied on the surface of PLA layer to create a 50 mm unbonded area, separating with the subsequent print layers during the printing process (Table 7B). Thereafter, the textile substrate, which was cut prior, to match the size of the build platform was placed above the blue tape separator, secured, and tensioned using binder clips to remove any wrinkles or crease. It is extremely important to position the clips carefully to prevent any obstruction in the path (top–bottom and both sides of the built platform). Afterwards, the printer was resumed to complete the print (Table 7C).

**Table 7:** The printing procedure involved in manufacturing ME polymer–textile composite (print layout A) to create T-Peel test panel

	1. First layer calibration for optimum Z-distance between build platform to extrusion nozzle 2. Print first or base PLA layer using 0.2 mm layer height 3. Add nozzle height increment while the first layer print was almost complete 4. Pause printer immediately once the first layer was completed
	5. Apply blue tape on the printed PLA layer
	6. Place and secure the textile substrate above the layers using binder clips 7. Resume the printer to complete the subsequent print layers
	8. Cut the T-Peel panel into six individual strips

The T-Peel test panel was printed as a whole sheet, then cut into six individual strips in 200 mm (L) × 25 mm (W) × 0.5 mm (H). Six T-Peel test panels for each polymer–textile combination were created, producing a total of 18 specimens to be tested. The unbonded area was pull separated by hand to form a “T” angle for the T-Peel specimen to be fixed to the top and bottom clamps on the testing machine for the T-peel test (Fig. 2). The adhesion value of the separated section will not be considered in the result as the unbonded area of the T-Peel test panel was designed to be clamped on the universal testing machine. Therefore, the net structure belonging to the upper or lower part of the unbonded area would not affect the adhesion result of this experiment.

### Test procedures

**Fig. 3:**
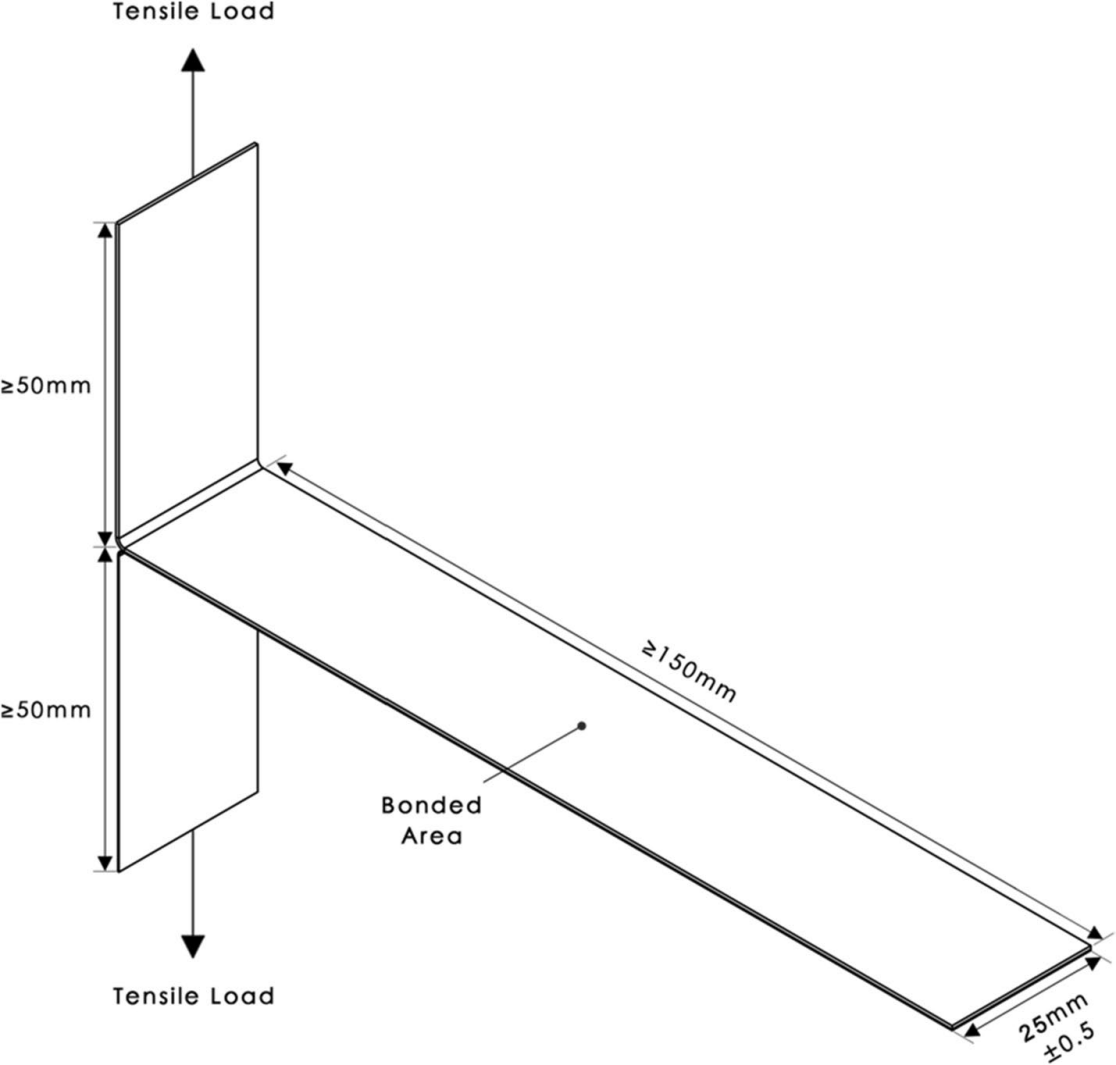
Technical drawing of the T-Peel test specimen in line with BS EN ISO 11339 (2010)

T-peel tests were carried out on the bonded ME printed polymer–textile composites to determine the peel force and peel strength required to separate the bonded polymers. Figure 3 shows a schematic diagram and dimensions of the bonded ME printed polymer–textile composites in line with BS EN ISO 11339 (2010).

All the specimens had the same nominal dimensions. The peel force was divided by the width (25 mm), to compute the peel strength. The tests were carried out using a universal testing machine (Instron 5969), which had a maximum load capacity of 500 N. The grips of the machine were used to secure the ends of the specimens so that they were subjected to uniform tension. The crosshead displacement was applied at a rate of 10 mm/min, based on the guideline given in BS EN ISO 11339 (2010). A digital camera was used to monitor the failure modes of the specimens.

## Results and discussion

**Fig. 4:**
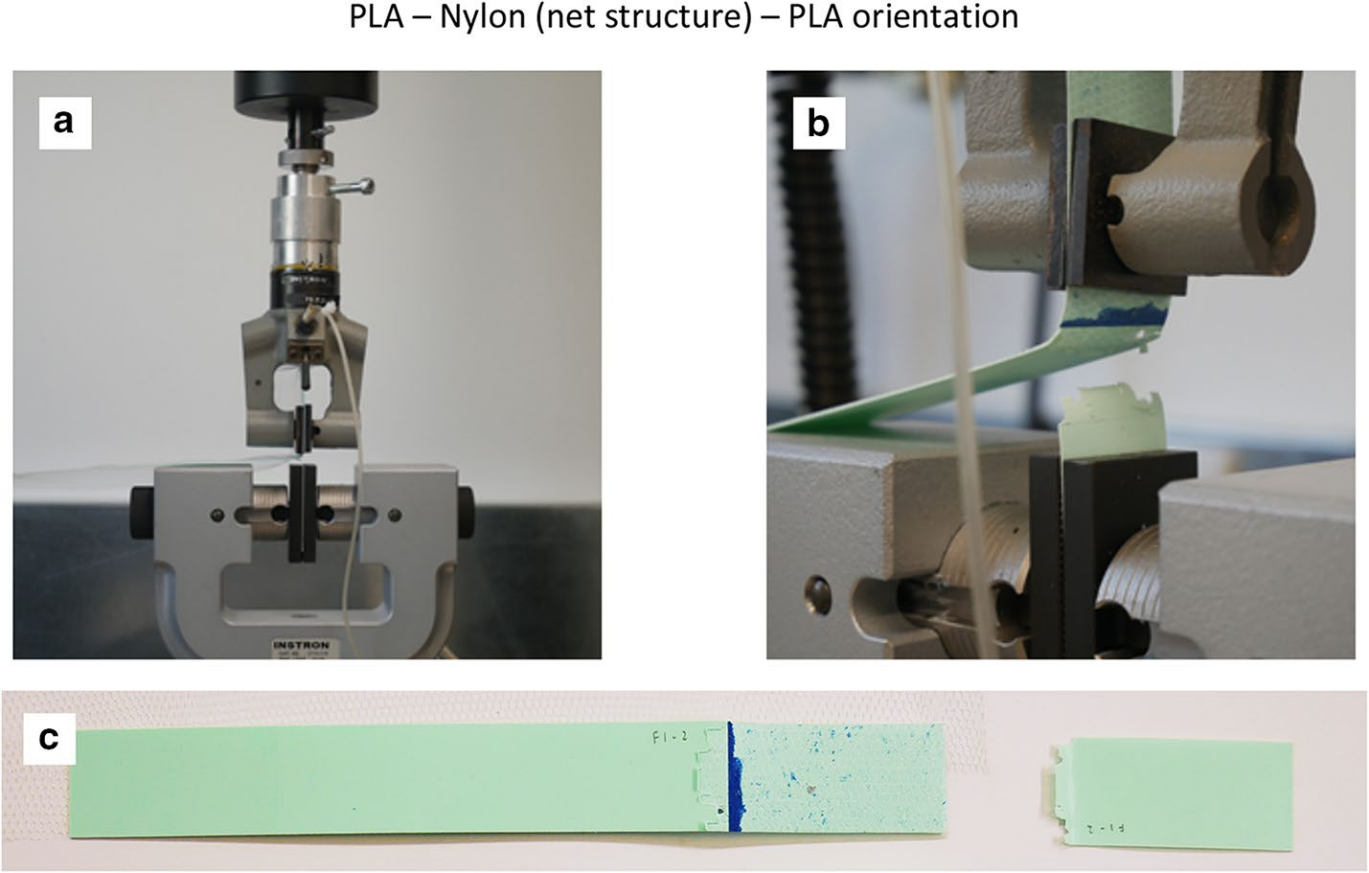
**a** Test setup, **b** failure mode for the PLA—Nylon (net structure)—PLA orientation and **c** failure of an adherend (cohesive substrate failure)

**Fig. 5:**
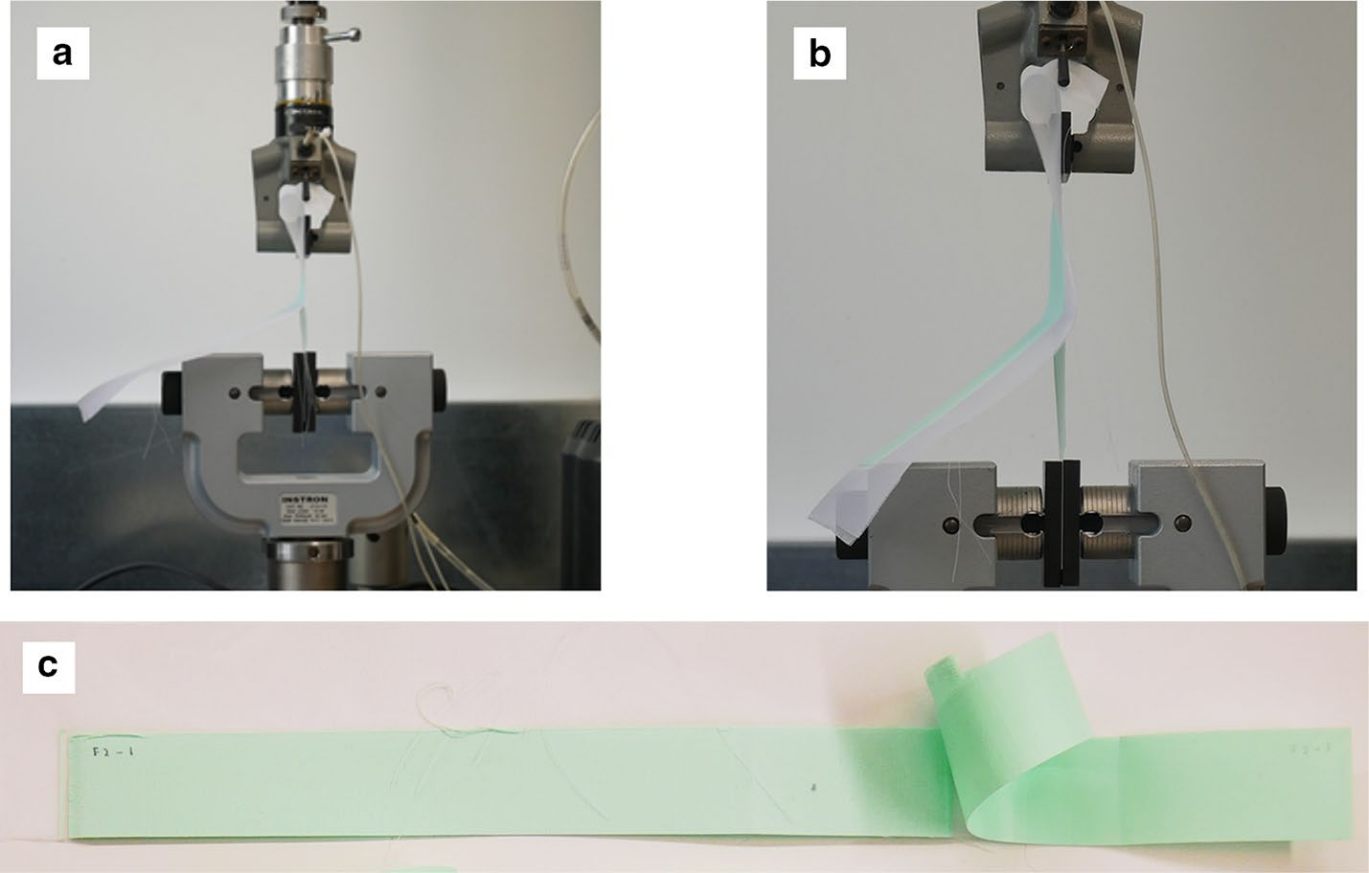
**a** Test setup, **b** failure mode for the PLA—polyester (voile structure)—PLA orientation and **c** adhesion failure (delamination)

**Fig. 6:**
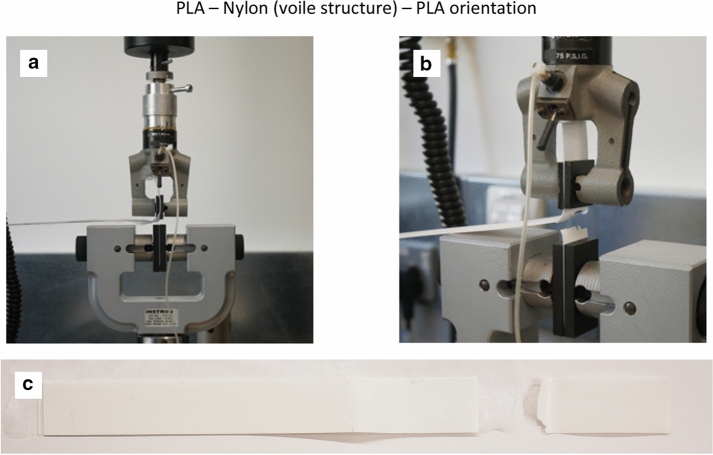
**a** Test setup, **b** failure mode for the PLA—Nylon (voile structure)—PLA orientation and **c** failure of an adherend (cohesive substrate failure)

PLA in general printed well on Polyester and Nylon textile substrate with good linear and haptic finishing. Due to a limited volume of turquoise coloured Prusa PLA filament, a white coloured Prusa PLA filament was used to create the remaining test specimens. The material properties remained the same whist the material colour would not have an impact on the T-Peel test results. Figures 4, 5 and 6 show the test setup on and failure modes for the PLA—Nylon (net structure)—PLA, PLA—Polyester (voile structure)—PLA and PLA—Nylon (voile structure)—PLA orientations, respectively. The failure mode classification was based on BS EN ISO 10365 (1995). For the two orientations with Nylon net structure (Fig. 4) and voile structure (Fig. 6), both were the failure of an adherend, caused by the fracture of printed PLA layer (cohesive substrate failure). On the other hand, the orientation with Polyester voile structure showed an adhesion failure mode, delamination of printed PLA layer (substrate) from the textile, shown in Fig. 5c.

**Fig. 7:**
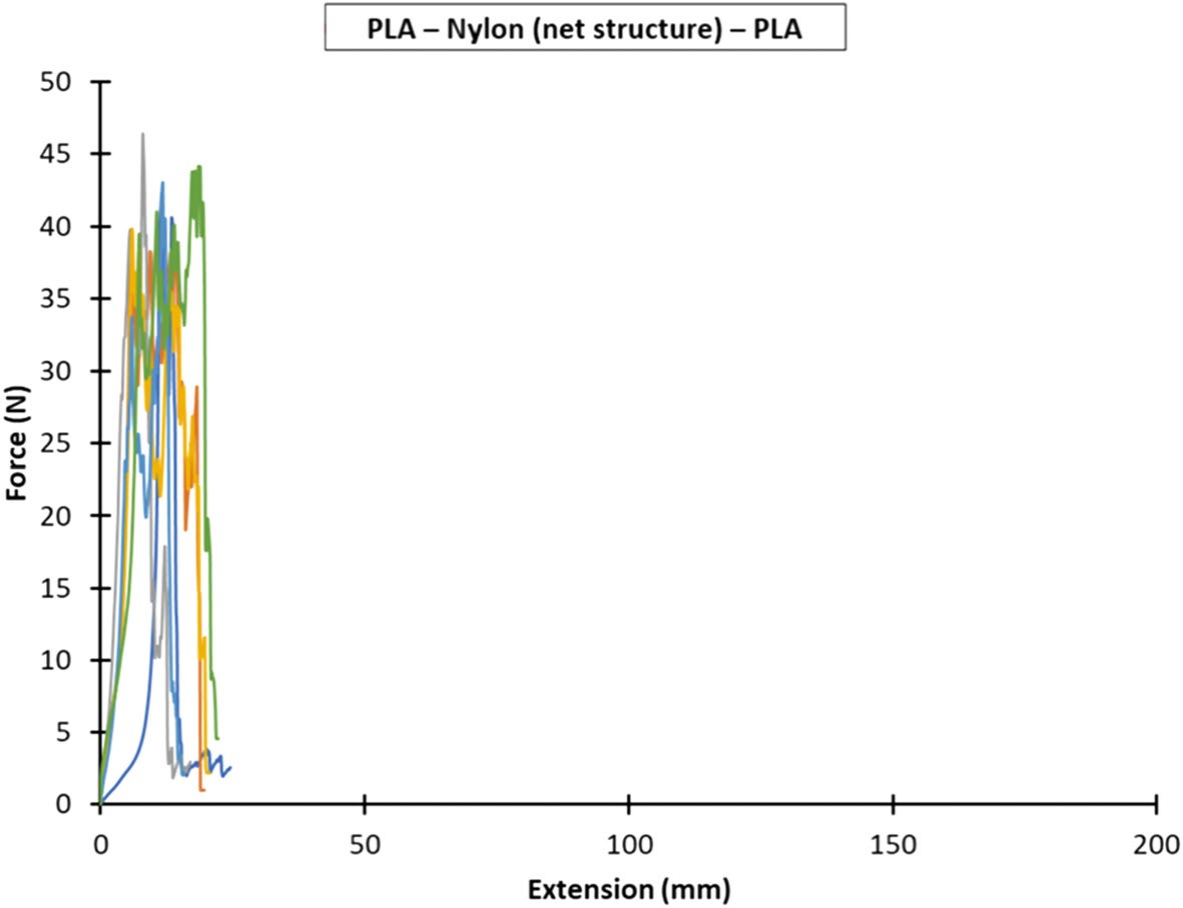
Force versus extension plots for six specimens with the PLA—Nylon (net structure)—PLA orientation

**Fig. 8:**
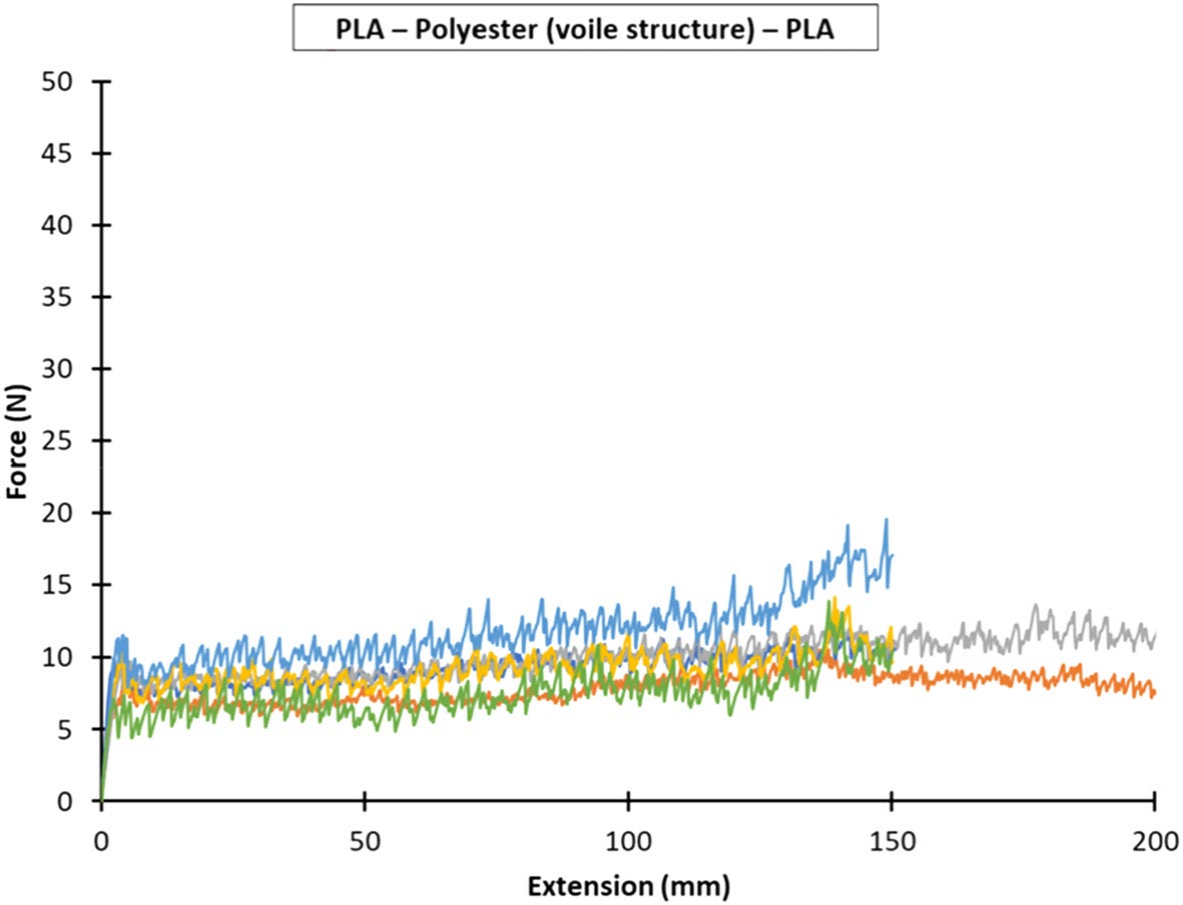
Force versus extension plots for six specimens with the PLA—Polyester (voile structure)—PLA orientation

**Fig. 9:**
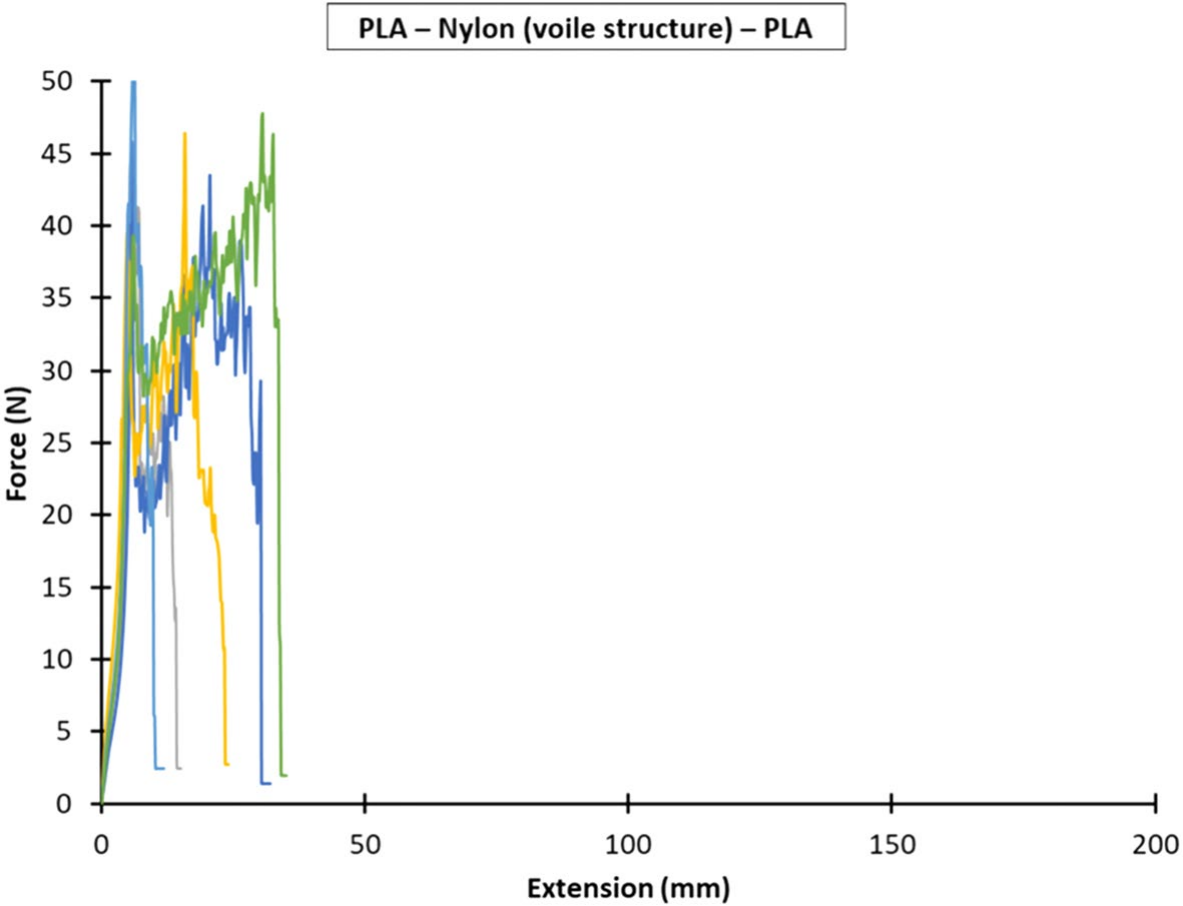
Force versus extension plots for six specimens with the PLA—Nylon (voile structure)—PLA orientation

**Fig. 10:**
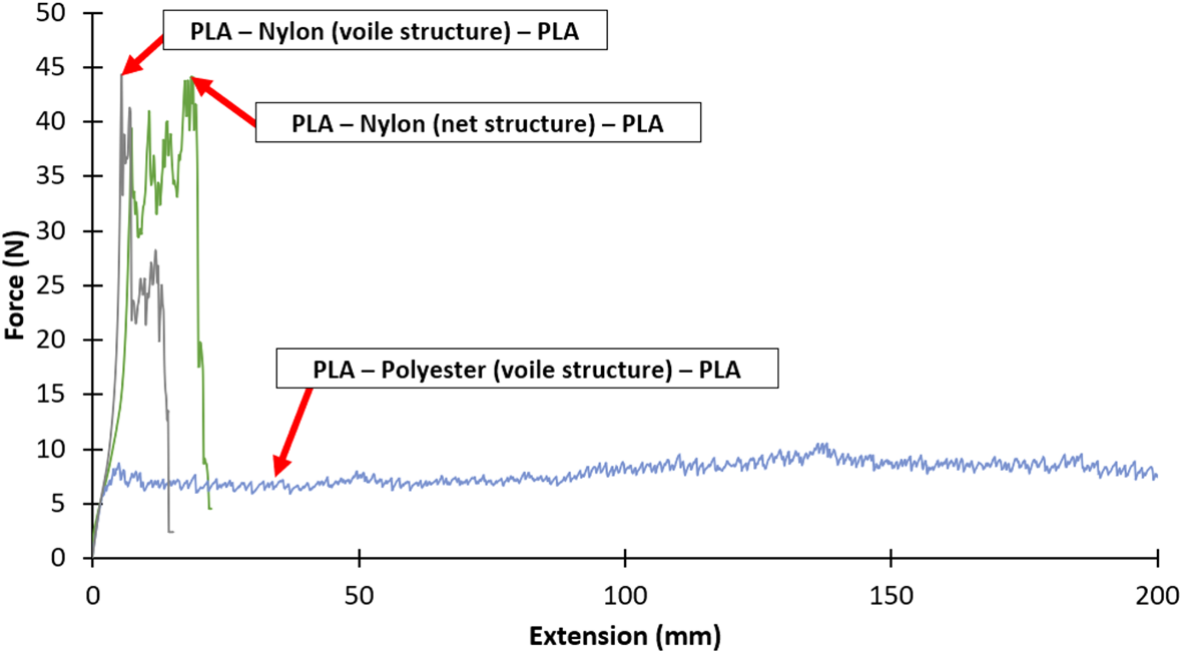
Comparison of the force versus extension plots for the three bonded ME printed polymer–textile composites

Figures 7, 8 and 9 show the force versus extension responses for the six specimens for the three different combinations of polymer–textile composites, (a) PLA—Nylon (net structure)—PLA, (b) PLA—Polyester (voile structure)—PLA, and (c) PLA—Nylon (voile structure)—PLA. For comparison, Fig. 10 shows a representative force versus extension responses for the three different combinations of polymer–textile composites. Similar to their failure modes, the force versus extension responses for PLA on Nylon net structure and voile structure were similar, far better results as compared to Polyester. For both PLA—Nylon composites, the initial force exceeded 40 N and included a few force peaks, up to a maximum extension of about 20 mm, which subsequently dropped leading to failure of the PLA polymer, reflecting a relatively stronger bond compared to PLA on Polyester textile.

In comparison, the force versus extension responses for the PLA—Polyester (voile structure) composite showed an initial linear response until about 7 N. After that, there were several force peaks (i.e. undulating curve), reflecting the gradual separation of the Polyester textile from the PLA polymer, until the end of the test. For all the PLA—Polyester (voile structure)—PLA T-Peel specimens, there was no damage to both printed polymer layer and textile substrate.

Following the recommendation given in BS EN ISO 6133 (2015), the average peel force was determined based on the midpoint of the minimum and maximum peak force values, whilst ignoring the initial rise at the start of the test. Table 8 gives the average peel forces, peel strengths and coefficients of variation for the three bonded ME printed polymer–textile composites. The peel strengths for the PLA—Nylon (net structure)—PLA, PLA—Polyester (voile structure)—PLA and PLA—Nylon (voile structure)—PLA polymer–textile composites were 1.3 N/mm, 0.4 N/mm and 1.4 N/mm, respectively.

**Table 8:** Average peel forces and strengths and coefficients of variation for the three bonded ME printed polymer–textile composites

Orientation	Peel force		Peel strength	
	Average (N)	Coefficient of variation (%)	Average (N/mm)	Coefficient of variation (%)
PLA—Nylon (net structure)—PLA	33.5	12	1.3	12
PLA—Polyester (voile structure)—PLA	9.5	18	0.4	18
PLA—Nylon (voile structure)—PLA	33.8	9	1.4	9

The results show that the average peel forces and strengths for both PLA—Nylon composites (net and voile structure) were about three times greater than PLA—Polyester composite (voile structure), which explained the breaking of the deposited layer at the beginning of extension in all samples during the T-Peel test. The statistical analyses show that the PLA—Nylon (voile structure)—PLA orientation had the lowest coefficient of variation of 9% and the PLA—Polyester (voile structure)—PLA orientation had a coefficient of variation of 18%, representing a relatively larger variation across the tested specimens.

PLA printed on Polyester textile did not show high peel strength result, which revealed that both materials were less compatible. According to the diffusion theory, the fine pore properties of voile structure decrease the amount of infiltration between the two polymer layers for polymer–polymer adhesion. As a result, the deposited polymer cannot protrude through the textile layer to create a form-locking connection (Eutionnat-Diffo et al. 2019; Sabantina et al. 2015; Unger et al. 2018). However, this theory was challenged when comparing the results obtained from both voile structures and the two PLA—Nylon composites. Despite both nylon textiles have different mesh structure (net and voile), weave type, thickness and pore sizes, there were no substantial differences on their peel force and strength. It can be concluded that the fibre type has a predominant effect on the interfacial bonding strength between the printing material and textile substrate due to the chemical nature of both and interpolymer polar interactions (Van der Waals dipole–dipole interactions) across phase boundaries as explained by Sanatgar et al. (2017). The compatibility between the printing material and the textile substrate fibre type has a significant effect on the polymer–textile adhesion. With respect to the textile stretchability, there was no direct and substantial effect of the textile stretch on the peel resistance, nevertheless, working with low level or non-stretch textile substrate improves the ease of printing process. It can be equally stretched in both vertical and horizontal directions to be secured on the build platform and no pre-strain to cause irregular pore circularity and pore area which will cause an inconsistent amount of infiltration of the printed polymer at the time of printing, which will correspondingly affect the peel strength (Narula et al. 2018).

Concerning the limitations of this study, the ME printing production process is demonstrated and tailored for Cartesian XZ hot end, Y bed ME desktop printer (i.e. Prusa i3 MK3S). Although the manufacturing concept is similar, there will be slight differences in the calibration and printing steps to accommodate the function of each ME printers, such as the Cartesian XY hot end, Z bed ME desktop printer (I.e. Ultimaker) (3D Printing Beta 2020). This study examined the mechanical and adhesion properties of ME polymer–textile composites using basic structure design to meet the standards’ requirement BS EN ISO 11339 (2010). Future work could explore printing different geometrical structures on more variety of textile substrates of different properties (i.e. weight and texture). PLA with different performance, mechanical properties and flexural characteristics mentioned in Table 2 can be explored. For instance, PLA Plus+ can be used for greater mechanical performance and resistance than regular PLA and has lower printing temperature compared to ABS and PETG (Gregurić 2020). Flexible PLA can be used to create soft and flexible prints that can drape according to the fluidity of the textile fabric (Griffin 2019).

## Conclusion

In this paper, the three key interconnected factors (the printing material, textile substrate, and printer settings) which affect the production, printed quality and adhesion strength of the polymer–textile composites were discussed. The experimental setup, procedures, and analysis techniques to quantify the adhesion properties of polymer–textile composites have been described, and the results were compared and discussed.

This study investigated the influence of varying textile substrate parameters (i.e. different fibre types, structure, and weights) on polymer–textile adhesion force. The printing material used, and ME printing parameters were kept constant. Different ME printed polymer–textile composites were manufactured using PLA (printing material) and Nylon and Polyester (textile substrates), to evaluate their manufacturing feasibility and assess their mechanical properties. The ME printed polymer–textile composites included (a) PLA—Nylon (net structure)—PLA; (b) PLA—Polyester (voile structure)—PLA; and (c) PLA—Nylon (voile structure)—PLA. Based on the results from the T-peel tests, it can be concluded that the compatibility between the printing material and the textile substrate fibre type has a dominant effect on the peel resistance of ME polymer–textile composite. The average peel forces and strengths for both printed PLA on Nylon textiles composites were nearly three times stronger than Polyester textile despite the differences in in their mesh structures, pore properties and weave type.

Finally, the work reported in this paper has not only added to the current limited knowledge of developing and testing of ME printed polymer–textile but also demonstrated and visualised the potential of direct ME of polymers onto textile fabrics as a material-joining technique for the development of new textile functionalisation and multi-material composite explorations. This new AM technique can exploit and promote new visions of personalised, localised and scalable garments with added functionalities, and boost the uptake of innovative and sustainable models for the textile industry. The principles and procedures uncovered from this study can be also applied for new applications or be extended to necessitate future research textile development.

## Data Availability

The datasets used and analysed during the current study are available from the corresponding author on reasonable request.

## Abbreviations

AM: Additive Manufacturing
BS: British Standards
FDM: Fused Deposition Modelling
FFF: Fused Filament Fabrication
FGAM: Functionally Graded Additive Manufacturing
ME: Material extrusion
PEI: Polyetherimide
PLA: Polylactic acid
PPE: Personal protective equipment
SPI: Stitches per inch
T_g_: Glass transition temperature
T_m_: Melt temperature
